# Biomechanical Analysis of Golf Swing Motion Using Hilbert–Huang Transform

**DOI:** 10.3390/s23156698

**Published:** 2023-07-26

**Authors:** Ran Dong, Soichiro Ikuno

**Affiliations:** 1 School of Engineering, Chukyo University, Toyota 470-0393, Japan; 2 School of Computer Science, Tokyo University of Technology, Hachioji 192-0982, Japan

**Keywords:** golf swing analysis, biomechanics, motion capture system, Hilbert–Huang transform

## Abstract

In golf swing analysis, high-speed cameras and Trackman devices are traditionally used to collect data about the club, ball, and putt. However, these tools are costly and often inaccessible to golfers. This research proposes an alternative solution, employing an affordable inertial motion capture system to record golf swing movements accurately. The focus is discerning the differences between motions producing straight and slice trajectories. Commonly, the opening motion of the body’s left half and the head-up motion are associated with a slice trajectory. We employ the Hilbert–Huang transform (HHT) to examine these motions in detail to conduct a biomechanical analysis. The gathered data are then processed through HHT, calculating their instantaneous frequency and amplitude. The research found discernible differences between straight and slice trajectories in the golf swing’s moment of impact within the instantaneous frequency domain. An average golfer, a single handicapper, and three beginner golfers were selected as the subjects in this study and analyzed using the proposed method, respectively. For the average golfer, the head and the left leg amplitudes of the swing motions increase at the moment of impact of the swings, resulting in the slice trajectory. These results indicate that an opening of the legs and head-up movements have been detected and extracted as non-linear frequency components, reviewing the biomechanical meaning in slice trajectory motion. For the single handicapper, the hip and left arm joints could be the target joints to detect the biomechanical motion that triggered the slice trajectory. For the beginners, since their golf swing forms were not finalized, the biomechanical motions regarding slice trajectory were different from each swing, indicating that beginner golfers need more practice to fix their golf swing form first. These results revealed that our proposed framework applied to different golf levels and could help golfers to improve their golf swing skills to achieve straight trajectories.

## 1. Introduction

In recent years, more detailed golf swing analysis has been conducted by using Trackman and high-speed cameras to quantify the golf swing. Trackman can quantify the angle of the club shaft during the golf swing and the spin of the golf ball [1]. In addition, the high-speed cameras employed in competitive golf broadcasts are capable of capturing a high-resolution 6000 fps video. However, these devices are quite expensive, making it impractical for average golfers to use them. Furthermore, amateur golfers may have difficulty in improving their golf swing using these devices because they require knowledge and experience in the golf swing. Therefore, in this study, frequency analysis is performed on the golf swing motion, and the causes of slice trajectories are visualized in a spectrum as an example, clearly revealing the wrong movements and helping golfers to improve their swing skills.

In general frequency analysis, Fourier transform (FT) and the Wavelet transform are commonly employed. These methods decompose signals linearly, so when non-stationary and non-linear data, such as human motion, are decomposed, it is necessary to expand to higher orders (about 200 modes), making interpretation from a biomechanics perspective difficult. On the other hand, the Hilbert–Huang Transform (HHT) is an analysis method that can capture physical features non-linearly [2]. When applied to human motion, HHT can decompose one motion into about six modes, easily interpreted from a biomechanical perspective, making it a suitable method for golf swing analysis. HHT applies Empirical Mode Decomposition (EMD) to decompose the time series data into several Intrinsic Mode Functions (IMFs) The IMFs are decomposed by assuming that the original data are formed by IMFs, which are pseudo-monochromatic waves, and one residual called Trend. Most important, each extracted IMF corresponds to a motion primitive [3]. The instantaneous frequency and amplitude of each IMF are calculated using the Hilbert Transform (HT). However, the method applied to golf swing analysis from a biomechanical perspective has not been conducted.

The purpose of this study is to quantify golf swing motion and identify movements of body parts that result in straight and slice trajectories in the frequency domain. The golf swing motion is quantified using inertial motion capture, called Perception neuron 2.0, which is relatively inexpensive and could be used in research [4,5,6]. Then, HHT is adopted for the collected golf swing data, and spectral analysis and biomechanics are discussed from the viewpoint of biomechanics. A spectral analysis is a method that the horizontal axis represents time, the vertical axis represents frequency, and the color represents amplitude. This method enables golfers to analyze their golf swing motion in the instantaneous frequency domain and present biomechanical details of golf swing motion analysis.

In this paper, Section 2 describes the methods adopted in this research by reviewing the related studies, discussing the biomechanics of the golf swing regarding straight and slice trajectories, introducing EMD and HHT, and proposing a flowchart of biomechanical analysis of golf swing using HHT. In Section 3, we apply our proposed method to golf swing motions collected in this study. We also demonstrate the results of an average golfer, a single handicapper, and three beginner golfers obtained using our method. Section 4 performs evaluations, including the sample size justification of golf swing data collection adopted in Section 3 and the sensitivity analysis of our proposed framework presented in Section 2. Section 5 analyzes and evaluates the results by discussing the spectra of our results shown in Section 3. Section 6 presents the conclusions obtained in this study.

## 2. Methods

### 2.1. Related Research

Golf swing movements are complicated since they require golfers to move their whole body, including almost all joints, within about 1–2 seconds. As a result, plenty of research has been conducted for tracking and analyzing golf swings. Watanabe et al. [7] proposed a measurement method for the driver’s head while swinging. Also, Nam et al. [8] performed a study to track golf swings using inertial sensors and a stereo camera. These studies demonstrated that motion capture systems and equipment could provide researchers to analyze golf swings based on kinematics. By using these systems, other research presented different golf motion systems to analyze golf swings [9,10]. Benefiting from these previous studies, countless researchers have presented more and more methods for improving golfers’ swing technique [11,12].

As we can see from these studies, biomechanics played an important role in improving golfers’ swing technique due to the complexity of human movements [13]. Consequently, research became more and more consecrated on analyzing golf swings from the biomechanical perspective [14,15]. Furthermore, studies were even deeper to investigate one part of the body, for example, the left arm or lumbar spine, during golf swings [9,16,17]. However, although the studies included above have been investing the golf swings from a biomechanical perspective [18], there is no research to conduct a biomechanical analysis of golf swings in the frequency domain.

Meanwhile, Huang et al. [19] presented a novel method named the empirical mode decomposition (EMD) and showed high performance in its Hilbert spectrum for analyzing non-linear and non-stationary time series data called the Hilbert–Huang Transform (HHT) in 1998. Although this method depends on experience instead of mathematical proof, its usefulness could also be confirmed [20]. Furthermore, studies focusing on mathematical proofs of EMD have also been conducted [21]. As a result, works to extend the usage of EMD were conducted continuously, from 2D image applications [22,23] to multivariate signal processing [24,25,26].

After HHT showed its high performance in analyzing non-linear time series data, Dong et al. [27] applied HHT to dance motions. Moreover, Dong et al. [3] also extended the usage for motions and proposed a novel framework to analyze human motions in the instantaneous frequency domain. These decomposed motions could be applied to robot motion design and deep learning [28,29], revealing its performance in the motion field.

However, for complicated motions like golf swing movements, in-depth analyses are required and have not been investigated yet. Thus, in this study, we apply HHT to golf swing motions and analyze the golf swings from the biomechanical perspective, providing novel knowledge in the frequency domain. Moreover, the golf swings are different for professional and amateur players [30]. Then, in this research, we also perform biomechanical analyses on golf swings in the frequency domain between different levels of golf players to provide comprehensive results.

### 2.2. Biomechanics of the Golf Swing

**Figure 1:**
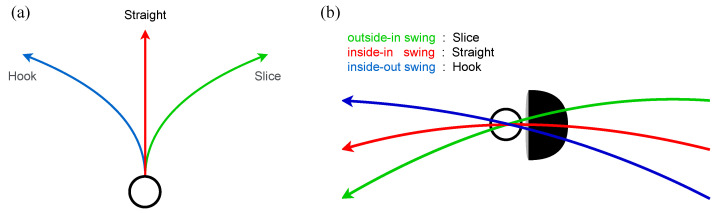
The three trajectory types of straight, hook, and slice. (**a**) Top view. (**b**) Side view with a golf club.

In this research, we investigate the trajectories of the golf ball to present biomechanical analyses in the frequency domain. There are roughly three types of ball trajectory in a golf swing, straight trajectory, hook trajectory, and slice trajectory, as shown in Figure 1a. The straight trajectory can only be obtained if the face of the golf club hits the ball perpendicular to the direction the ball travels, indicated in Figure 1b.

**Figure 2:**
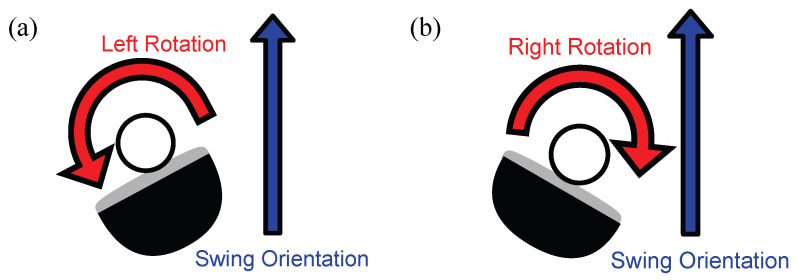
The mechanism of causing diffident types of trajectory. (**a**) Rotate left (hook). (**b**) rotate right (slice).

A hook trajectory is a trajectory in which the face of a golf club hits, and the ball is rotated to the left and curves to the left, as shown in Figure 2a. On the other hand, the trajectory in which the face of the golf club hits and the ball rotates to the right, as shown in Figure 2b, and the trajectory flies to the right while shooting is called the slice trajectory.

**Figure 3:**
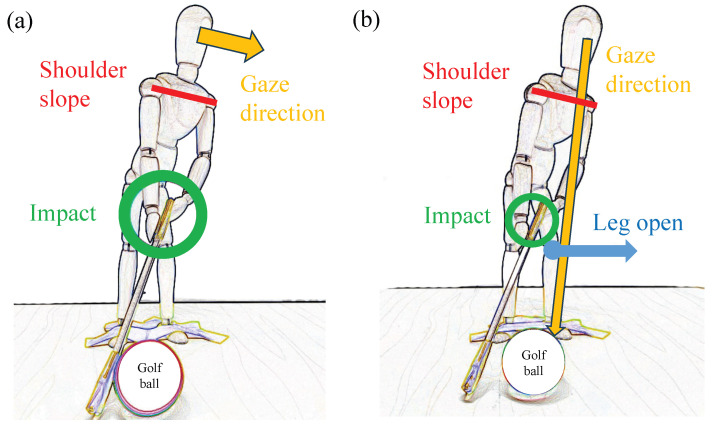
The biomechanics of causing slice trajectory. (**a**) Head-up motion. (**b**) Open body at impact.

Generally, a straight trajectory is desired in amateur golf. However, it is difficult for amateur golfers to achieve a steady straight trajectory, which often results in a hook or slice trajectory. In particular, it is very difficult for amateur golfers to improve their swings to produce a slice trajectory. The major causes of a slice trajectory are a head-up motion during the swing and a body opening motion in which the chest and front foot axis face in the direction of the ball just before impact. A head-up motion is a motion in which the line of sight is directed in the direction of the ball just before impact, as indicated by the yellow line in Figure 3. In addition, the head-up movement is also classified as a motion when the head is raised without being able to maintain the forward-leaning posture until the impact.

Due to these head-up motions, the upper body rises just before the impact, as shown in the red line in Figure 3a, and the arm swings behind the upper body, as shown in the green line in Figure 3a. The opening of the face of the golf club is induced, resulting in a slice trajectory.

On the other hand, the motion of opening the body is the motion in which the person’s front chest and the knee of the front leg axis face the direction of the ball before impact, as shown by the red and blue lines in Figure 3b. This motion is also similar to the green circle in Figure 3b because the arm swings behind the upper body. The opening of the face of the golf club is induced, resulting in a slice trajectory.

Thus, it is difficult for an amateur golfer to identify the head-up motion and the open body at impact just before impact, even if the swing is photographed and checked. It is also very difficult to identify which body part is affected at the best time. Therefore, in this study, we focus on the joints related to the head-up motion and the open body at impact before impact, which are the causes of the slice trajectory.

### 2.3. Hilbert–Huang Transform

HHT decomposes a signal into multiple IMF by EMD and then applies HT to the decomposed IMF to analyze its time-frequency characteristics. EMD is suitable for analyzing non-stationary and non-linear signals. In EMD, the input signal x∈R is assumed to be formed by multiple oscillation modes called the Intrinsic Mode Functions (IMF) and a residual *r* called a trend. That is, the signal *x* is defined as follows [2]

(1) $$x\left(t\right)=\sum_{i=1}^{n}{\mathrm{IMF}}_{i}\left(t\right)+r\left(t\right)$$

where $\sum_{i=1}^{n}{\mathrm{IMF}}_{i}\left(t\right)$ indicates the set of intrinsic mode Functions, r(t) indicates the residual. *n* indicates the number of decomposed IMF. The decomposed IMF has the same number of extreme values and zero crossings, and the oscillations are symmetric with the local mean. The IMF can be defined as follows [2]:

- The number of extreme values and zero crossings are equal or have at most one difference in the whole data.
- At any point, the mean value of the envelope connecting the local maximum and the local minimum is zero.

To obtain IMF by EMD, all local maxima and minima are specified for the data, and an envelope is created using a cubic spline function. The mean value of the created envelope is $m_{1}$, and the difference from the original data, $h_{1}$, is defined as follows:

(2) $$h_{1}=x\left(t\right)-m_{1}$$

The $h_{1}$ obtained by this operation is not symmetric and does not satisfy the definition of IMF. Therefore, $h_{1}$ is processed in the same way as $h_{2},h_{3}\cdots h_{k}$ in order to approach the definition of IMF where *k* indicates the iteration. After repeating the sieving up to *k* times, $h_{1k}$ satisfies the definition of IMF, as shown in the following:

(3) $$h_{1k}=h_{1(k-1)}-m_{1k}$$

(4) $${\mathrm{IMF}}_{1}=h_{1k}$$

The first IMF is extracted as IMF. To determine if IMF satisfies the definition, convergence conditions must be set [20]. Here, using Cauchy’s convergence judgment method, the convergence condition SD is:

(5) $$SD=\frac{\sum_{t=0}^{T}\;{\left[h_{k-1}\left(t\right)-h_{k}\left(t\right)\right]}^{2}}{\sum_{t=0}^{T}h_{k-1}{\left(t\right)}^{2}}$$

If SD is less than a predetermined value, the iterative process is stopped. The same process is repeated until all IMF are extracted.

However, golf swing motions were obtained as a multivariate signal by several motion sensors. Therefore, in this study, multivariate empirical mode decomposition (MEMD) is employed for analysis.

After decomposing the signal into multiple IMFs and trends using MEMD, the instantaneous frequency and amplitude are obtained for each IMF using HT. This series of processes is called HHT. In HHT, the analytical signal IMF is assumed to consist of a real part IMFiRe and an imaginary part IMFiIm, and is defined as follows

(6) IMFi(t)=IMFiRe(t)+jIMFiIm(t)

where IMFiRe denotes IMF decomposed by EMD from the original data as (1), and IMFiIm denotes the imaginary part obtained by HT using (7) [31]. Here, *j* denotes $\sqrt{-1\;}.$:

(7) IMFiIm(t)=1πPV∫−∞∞IMFiRe(τ)t−τdτ

Here, the PV indicates the Cauchy principal value. Then, using the obtained IMFiRe and IMFiIm, the instantaneous amplitude $A_{i}$ and instantaneous frequency $\omega_{i}$ of each IMF can be obtained as follows:

(8) Ai(t)=IMFiRe(t)2+IMFiIm(t)2

(9) ωi(t)=dθdt=ddttan−1IMFiIm(t)IMFiRe(t)

In this study, we calculate instantaneous frequencies and amplitudes from several captured golf swings of different level golfers to present a biomechanical analysis in the frequency domain

### 2.4. Proposed Flowchart of Biomechanical Analysis of Golf Swing Using HHT

**Figure 4:**
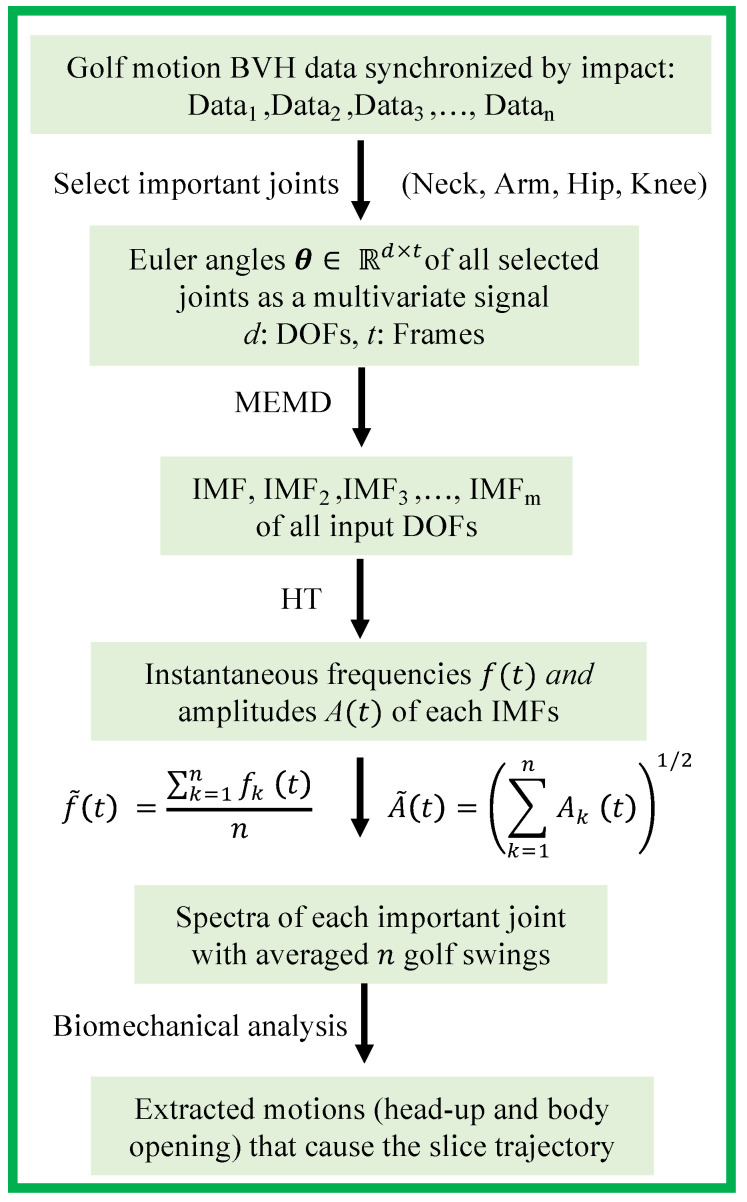
Biomechanical analysis flow chart to detect trigger motions for slice trajectory.

In this study, we propose a biomechanical analyzing framework for captured data of golf swing motions using HHT based on the previous research [3]. Figure 4 indicates the proposed analysis flow chart for the golf swing to detect and extract the biomechanical motion that causes the slice trajectory. Our analysis flow can be demonstrated as follows:

1. Three Euler angles, θx, θy, and θz, are prepared for 59 joints of the whole body in the golf swing motion. In this study, three types of swings are sampled: a swing in which the ball flies straight ahead and two swings that slice to the right (head-up and body-opening).
2. To ensure the correct features can be obtained, several golf swings of one person need to be motion captured and synchronized by the impact point.
3. Input channel data, all swing data with important joints that are represented by Euler angles θx, θy, and θz, into MEMD to obtain multivariate golf swing IMFs.
4. Apply the HT to each IMF to obtain the instantaneous frequencies and instantaneous amplitudes.
5. The average of the instantaneous frequency and amplitude of each swing is obtained as shown in Figure 4.
6. The Hilbert spectrum is created from the frequencies and amplitudes calculated from the above methods.
7. Analyze the biomechanical motions that caused the slice trajectory.

**Figure 5:**
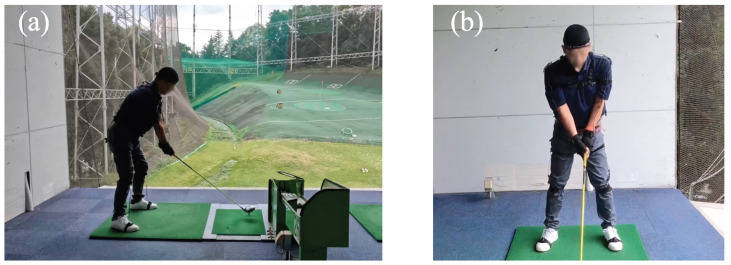
Golf swing motion capturing using Perception Neuron 2.0 of an average golfer with 10 years of golfing experience. (**a**) Back view. (**b**) Side view.

**Figure 6:**
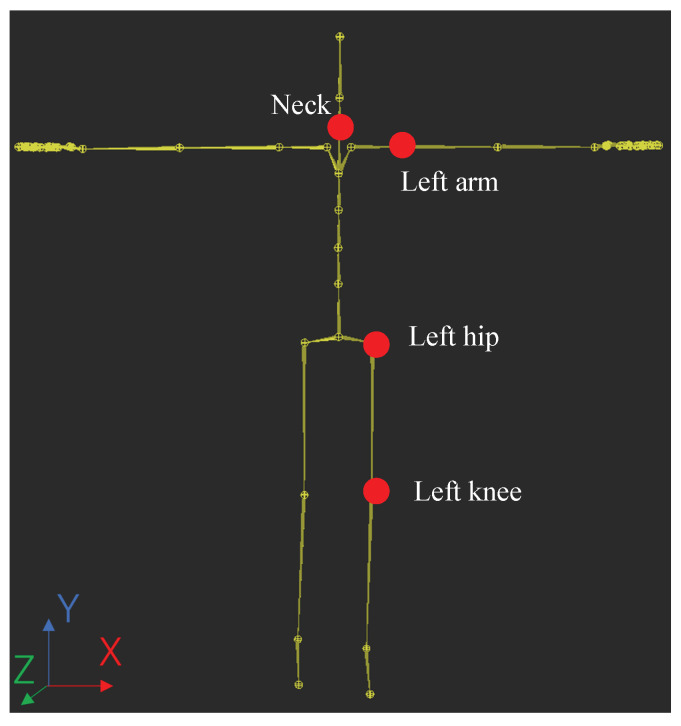
The T-pose definition of the skeleton model adopted in this study. Red points indicate the most important joint in causing the slice trajectory of the golf swing motion. (The skeleton model is made using MotionBuilder2023).

In total, 17 accelerometers were attached to the points to capture swing motion data using Perception Neuron 2.0, as shown in Figure 5. The collected data include the Euler angles $\theta_{x}$, $\theta_{y}$, and $\theta_{z}$, and the data are recorded at a sampling rate of 120 Hz. These data are projected into a hierarchical skeleton structure on the 59 joint balls of the whole body from the obtained rotation angle data of 17 sensors. Figure 6 shows the skeleton model with the origin pose as T-Pose.

The input motion datum is a Biovision Hierarchy (BVH) file, which is a file format that describes a skeleton model in a hierarchical structure with each joint. And each joint of the body moves with three degrees of freedom (Euler angles) θx, θy, and θz. Figure 6 demonstrates a BVH file that was adopted in this study. Since each joint can be presented as three Euler angles, the Euler angle can be understood as the angle of rotation of three successive rotations as described above, and it can be obtained directly from the orientation of the axes of the coordinate system. Although Euler angles have the problem with the uniqueness of solutions and gimbal locks, there are also studies to resolve the problem in order to use the Euler angle [32,33].

As shown in Figure 6, comparisons are made by focusing on the most important joints, the head (Neck) and left half of the body, left arm, left hip (LeftupLeg), and left knee (LeftLeg), rather than comparing all 59 joints.

Furthermore, the most important thing is that since the Euler angles are humanly interpretable that are different from the quaternion, the results obtained by our method could help golfers easily understand unnecessary movements and improve their golf swing skills. For example, Wheare et al. [9] conducted research about golf swings using the joint angular to describe the human body, presenting intuition knowledge on human body movements. Thus, in this study, we still adopt BVH data to analyze golf swing motions. To make sure that the gimbal locks did not occur, we compared the collected skeleton data with the original video before the analysis. Furthermore, we also confirmed that if the original data are correctly digitized before inputting them into our analysis framework, ensuring we can obtain the expected HHT spectra.

For the BVH motion files adopted in this study, their Euler angles were all normalized using software called MotionBuilder, as shown in Figure 6. MotionBuilder is commonly employed in the motion research fields to visualize, analyze, edit, and normalize motion data in different areas [34,35,36]. Then, in this study, we normalize all captured golf swing data collected by the motion capture system in an Euler order θz−θx−θy using MotionBuilder, which the order was also adopted in the previous research [37].

However, the gimbal locks may occur in the motion data, especially in the shoulder joints, which are very complex joints during IMU measurements [38], although MotionBuilder has a filter called Gimbal Killer [39] and already has been employed in different areas. Thus, in this study, we mainly focus on the neck joints that correspond to the head-up motion and the knee joints that correspond to the open body at impact to reveal the biomechanical movement triggering the slice trajectory. Furthermore, as shown in Figure 4, we employ the Euclidean metric to deal with the decomposed Euler angles to investigate the intensity of each joint. Thus, in the unlikely event of gimbal locks, our results shown in the spectra can still be considered less affected by the gimbal locks.

**Figure 7:**
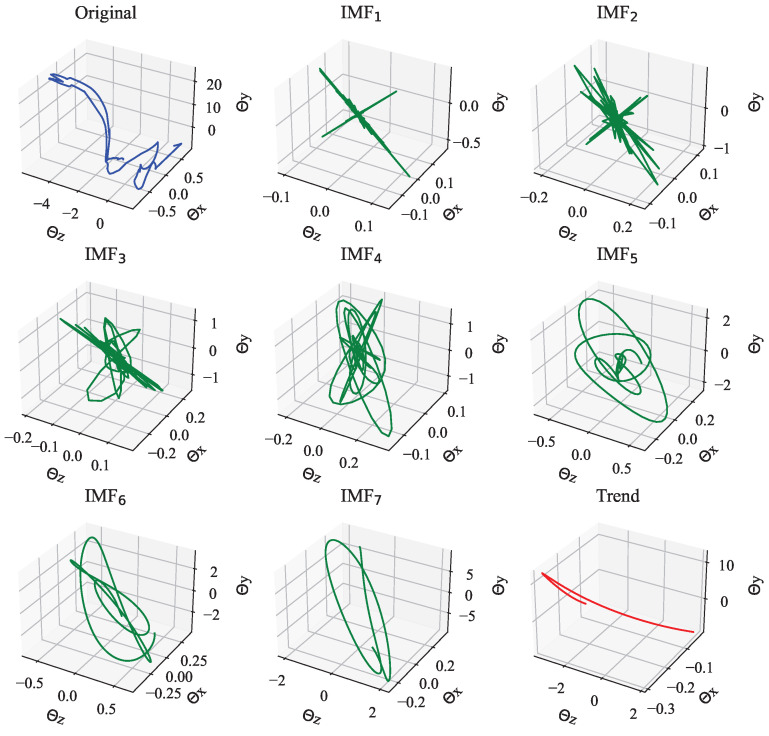
An example of decomposing Euler angles of the neck joint selected from one golf swing motion data (blue) into several IMFs (green) with a trend (red) using MEMD.

Figure 7 shows the decomposed results of the neck joints selected from one golf swing motion data. As can be seen from Figure 7, the Euler angles θx,θy,θz in the original are changing in three-dimensional space. To show an example of the decomposition of these Euler angles that are considered as a multivariate signal to be decomposed by MEMD, we illustrate a decomposition example with the original one as Figure 7. In this example, Figure 7 demonstrates that 7 IMFs with one trend have been extracted from the original data and all Euler angles θx,θy,θz were, respectively, decomposed.

After obtaining the IMFs, we apply HT to each IMFs to calculate instantaneous frequencies and amplitudes according to (8) and (9). To better understand biomechanical motion as one joint instead of three Euler angles, we calculate the Euclidean metric of three instantaneous amplitudes A(t) since the Euler angles θx,θy, and θz are orthogonal to each other, and we also average the instantaneous frequencies ω(t) due to the three Euler angles data come from the same signal source based on HHT property [2]. Then, we can obtain a spectrum of one joint from only one sample.

To verify our biomechanical analysis method, we captured several golf swings with straight and slice trajectories from an average golfer and a single handicapper with golf experience, and three beginner golfers without experience, respectively. Then, we adopted these data into our method to demonstrate the biomechanical movements causing the slice trajectories.

## 3. Results

As described in Section 2, golf swing motions are quantified by inertial motion capture and analyzed in the instantaneous frequency domain by applying Hilbert–Huang transform to the data. The causes of the body motion that results in a slice trajectory and a straight trajectory are examined from the viewpoints of biomechanics based on spectral analysis.

To demonstrate our method, in this study, we collected the golf swing data of an average golfer with 10 years of golfing experience, a single handicapper with 15 years, and three beginner golfers with almost no experience. However, for simplicity, we mainly discuss the results according to 10 years of golfer’s data with the 1-Wood golf club in this paper, and apply the single handicapper and the three beginner golf data for verification.

### 3.1. Average Golfer Experiment

In order to compare swings with a slice trajectory and swings with a straight trajectory, data from 6 straight trajectory swings, 6 slice trajectory swings with a head-up motion, and 6 slice trajectory swings with an open body motion are adopted for this analysis. These data were collected from an average golfer, as shown in Figure 5.

Table 1 shows the swing time, the total number of frames, and the number of frames of impact timing for each swing pattern after trimming. In order to ensure our results and discussions are correct, the average of instantaneous frequencies and amplitudes of the 6 swings taken for each of the straight, slice (head-up motion), and slice (open body at impact) types is calculated after swing data decomposed by MEMD and extracted IMFs applied by HT, according to the proposed analysis framework shown in Figure 4.

**Figure 8:**
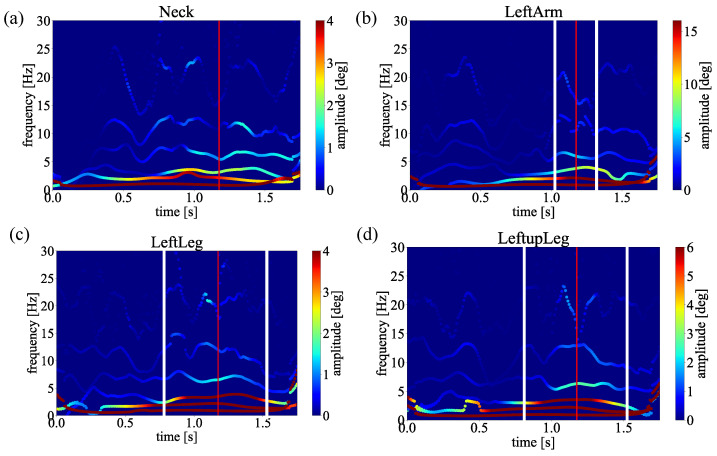
The HHT spectrum analysis of the straight trajectory. (**a**) Neck. (**b**) Left arm. (**c**) Left hip. (**d**) Left knee.

**Figure 9:**
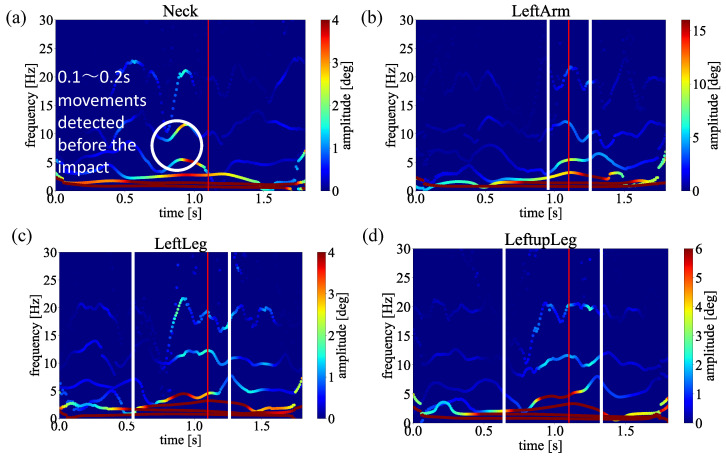
The HHT spectrum analysis of the slice trajectory caused by head-up motion. (**a**) Neck. (**b**) Left arm. (**c**) Left hip. (**d**) Left knee.

**Figure 10:**
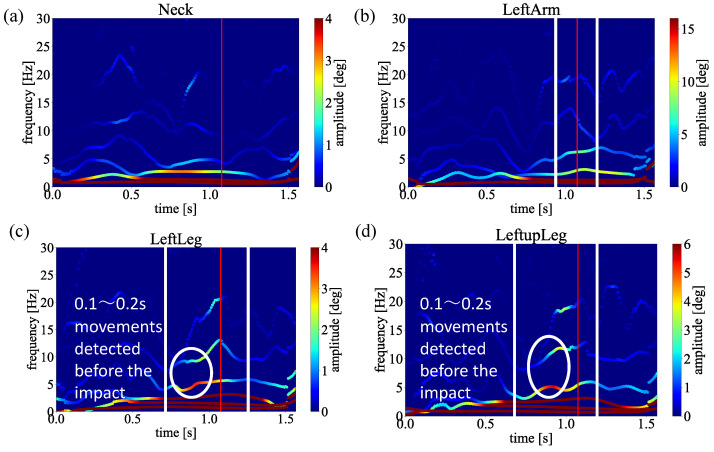
The HHT spectrum analysis of the slice trajectory caused by open body at impact. (**a**) Neck. (**b**) Left arm. (**c**) Left hip. (**d**) Left knee.

Since the averaged frequency and amplitude could present non-linear physical properties [40], to present comprehensive and credible results, we average the spectra obtained from 6 samples shown in Table 1. When calculating the average, the impact timing also differs because the swings’ speed differs depending on the data. Therefore, the data size was adjusted to the smallest data size centered on the impact timing. Finally, the obtained spectra are shown in Figure 8, Figure 9 and Figure 10. Notice that the Euler angles decomposed by MEMD and transformed by HT, respectively. The average was only taken in the spectral analysis process.

As a result of applying MEMD to these data, each data was decomposed into 6 IMFs. Spectral analyses for different groups are performed by applying HT to each decomposed IMF and obtaining the averages of each joint. The obtained results are represented by time on the horizontal axis, frequency on the vertical axis, and amplitude on the color. The results of each averaged swing are shown in Figure 8, Figure 9 and Figure 10. Here, the red lines in the figure indicate the timing of the impact point. The moment of impact. Ideally, the amplitude of the frequency should be high (red) at the moment of impact. There should be no unnecessary movement before and after the impact. Moreover, the best distribution is a pendulum-like throw-in and throw-out with amplitude centered on the impact, as shown by the white line. By comparing Figure 8, Figure 9 and Figure 10, only straight trajectory has the best distribution in the most important joints biomechanically regarding the slice trajectory.

As demonstrated in the previous research, the resolution of the Perception Neuron 2.0 is 0.02 [deg]. The static accuracy is Roll: <1 [deg], Pitch: <1 [deg], and Yaw angle: <2 [deg], which are corresponding to Euler angles $\theta_{x}$, $\theta_{y}$, and $\theta_{z}$ [41]. In contrast, the smallest motions detected in the spectra are also bigger than 10 [deg], indicating that errors due to the motion capture equipment could be ignored during our analysis. That is, the output of our method can provide correct advice to golfers using this IMU motion capture system.

### 3.2. Single Handicapper Experiment

**Figure 11:**
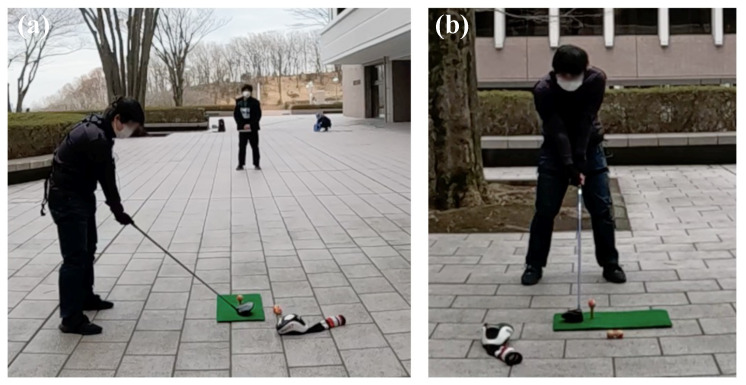
Golf swing motion capturing using Perception Neuron 2.0 of a single handicapper with 15 years of golfing experience. (**a**) Back view. (**b**) Side view.

Next, to verify our proposed method can be adopted to analyze the golf swing based on the biomechanical perspective in the same way as the results obtained above, we also apply our framework to a signal golfer swing motion. Figure 11 shows a signal golfer swing motion experimented upon under the same circumstances. The data were also averaged by six swings for each straight and slice trajectory to present a comprehensive result.

**Figure 12:**
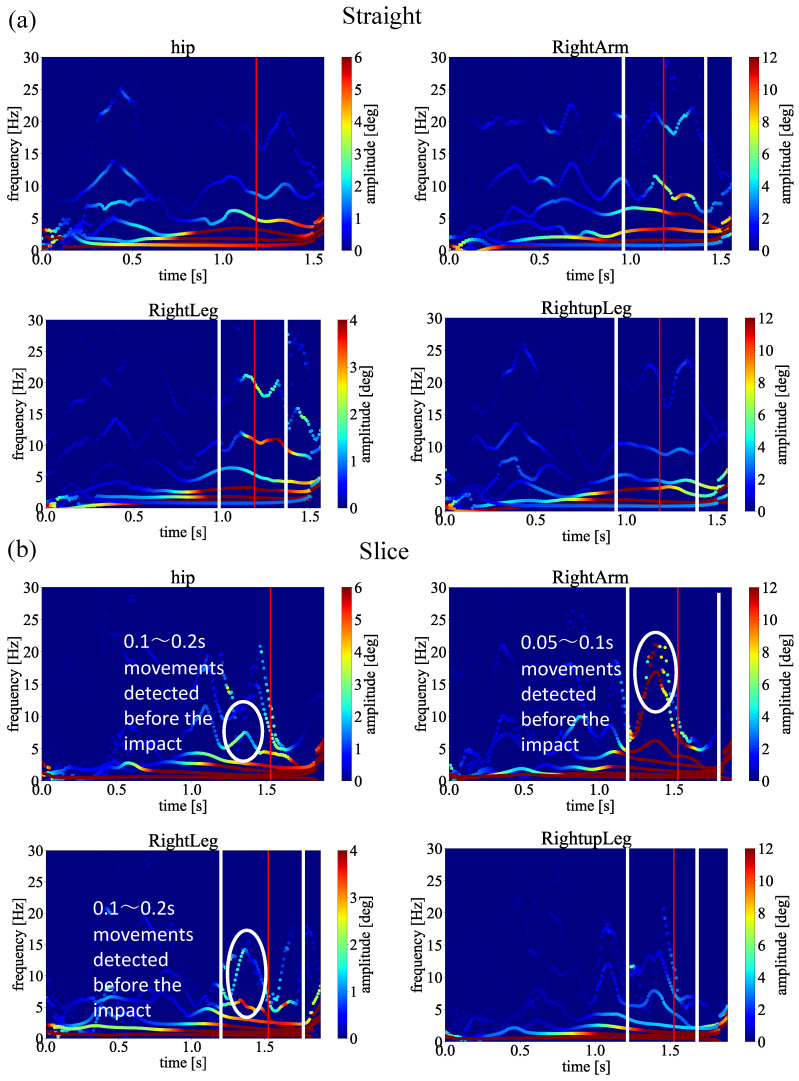
The HHT spectrum analysis of the single handicapper swing motion. (**a**) Straight trajectory. (**b**) Slice trajectory.

Figure 12a,b shows the spectrum analysis of the single handicapper swing motion. For simplicity, we only demonstrate the results between straight and slice trajectories in the following verification experiments. As shown in Figure 12b, the same movements have been observed in the slice spectrum just before the impact points, indicating there is an unnecessary movement triggering the slice trajectory, while there is no such movement in Figure 12a. These results show our proposed method could be applied to single handicappers.

### 3.3. Beginner Golfer Experiment

Since we only demonstrate an average golfer and a single handicapper with experience as examples, the analysis of the beginners without experience is also needed to test whether our method can provide a biomechanical analysis for the golf swings. In this study, we also apply our framework to three beginner golfers who have yet to gain golf experience. To demonstrate and verify that our proposed golf swing biomechanical analysis method can be adopted for different golfers, we apply the flow chart shown in Figure 4 to the three golf swing motion data. The spectra have been divided into two groups. One group is golf swing motions with a straight trajectory. Another group is golf swing motions with a slice trajectory. They have been averaged from several swing motions, respectively, motions of the average golfer and signal golfer experiment. The results of the biomechanical analysis with spectra illustrate all figures shown below. The red lines also indicate the impact point.

**Figure 13:**
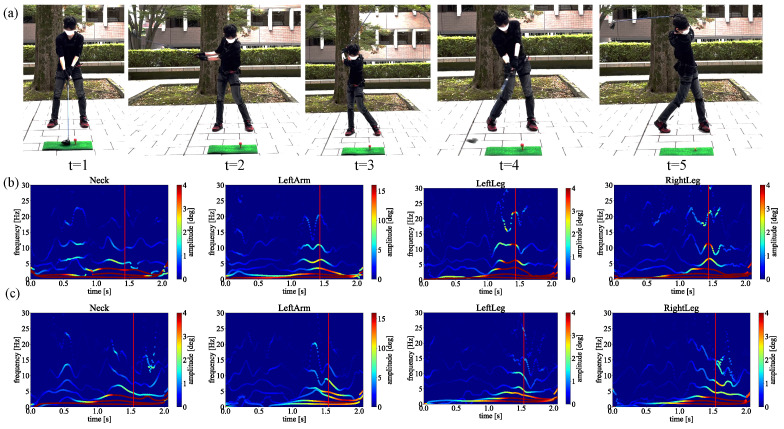
The HHT spectrum analysis of the beginner golfer 1 without golf experience. (**a**) Golf swing motion capturing using Perception Neuron 2.0. (**b**) Straight trajectory. (**c**) Slice trajectory.

Figure 13a–c shows our framework results for one of the beginner golfers experimented with in this study. As Figure 13a shows, we also adopt the same equipment to experiment on the beginner golfer to keep that we can obtain the motion data under the same circumstances.

**Figure 14:**
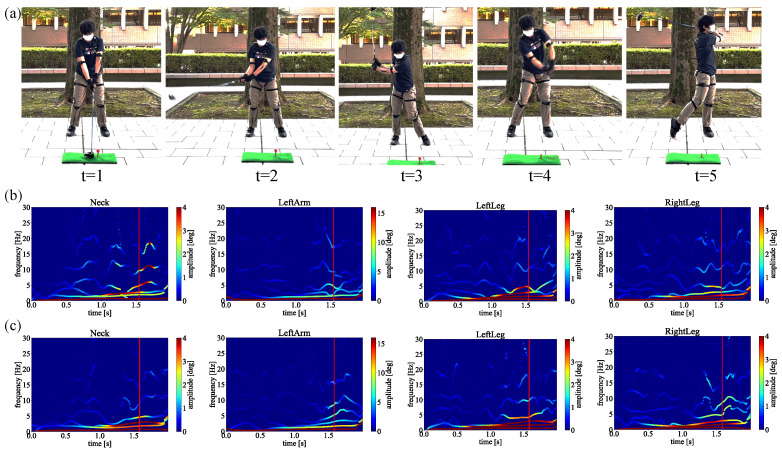
The HHT spectrum analysis of the beginner golfer 2 without golf experience. (**a**) Golf swing motion capturing using Perception Neuron 2.0. (**b**) Straight trajectory. (**c**) Slice trajectory.

Figure 14a–c shows that we adopt the same motion capture system to obtain other beginner golf swing motion data. To analyze the motion from the biomechanical perspective by our proposed method, we perform the spectrum analysis of each straight and slice trajectory after averaging by several swings, as shown in Figure 14b,c.

**Figure 15:**
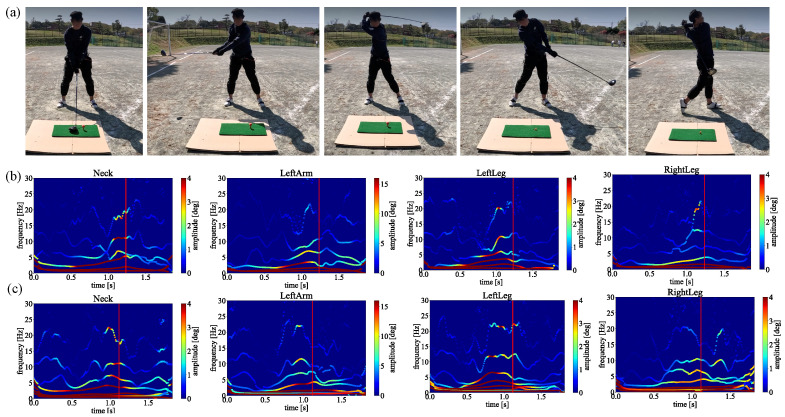
The HHT spectrum analysis of the beginner golfer 3 without golf experience. (**a**) Golf swing motion capturing using Perception Neuron 2.0. (**b**) Straight trajectory. (**c**) Slice trajectory.

Figure 15a–c, we also adopt the same motion capture system to obtain the third beginner golf swing motion data. Also, to give comprehensive results, we captured the swing data and averaged them by several swings for each straight and slice trajectory, as same as the single golf we showed above.

## 4. Evaluations

In this section, we indicate that our results shown in the above sections were valid and that our proposed frameworks can be adopted in the golf swing teaching system. In addition, the sample size justification and sensitivity analysis are performed and discussed in this section.

The mean value and standard deviation (SD) with the coefficient of variation (CV) are adopted in the sample size justification section, and the decomposition experiments with different joint inputs are discussed with MEMD property in the sensitivity analysis section.

### 4.1. Sample Size Justification

Table 2 shows the mean values and SD with CV for instantaneous frequency obtained by averaging six swings with each subject. In this research, we captured five subjects, with each six swings for each street and slice trajectory. To demonstrate the six swings number is enough for our analysis, we show the neck joint averaged data obtained from the straight swings at Table 2.

As we can see from the table, the mean value and SD decrease as the number of IMFs increases. Since we took the average from the six swings, the CV value that indicates the data variability could be a quantitative indicator to verify the sample size. In Table 2, all CV values are under 1, which means the results we used in the above sections have less variability since they were averaged from the six swings. Furthermore, the IMF${}_{2}$, IMF${}_{3}$, and IMF${}_{4}$ have the smaller CV than others. On the other hand, we nearly adopted the IMF${}_{2}$, IMF${}_{3}$, and IMF${}_{4}$ to demonstrate the results shown in Figure 9, Figure 10 and Figure 12. Thus, these results indicate that the sample size adopted in the present research is enough to analyze and validate our proposed method.

### 4.2. Sensitivity Analysis

**Figure 16:**
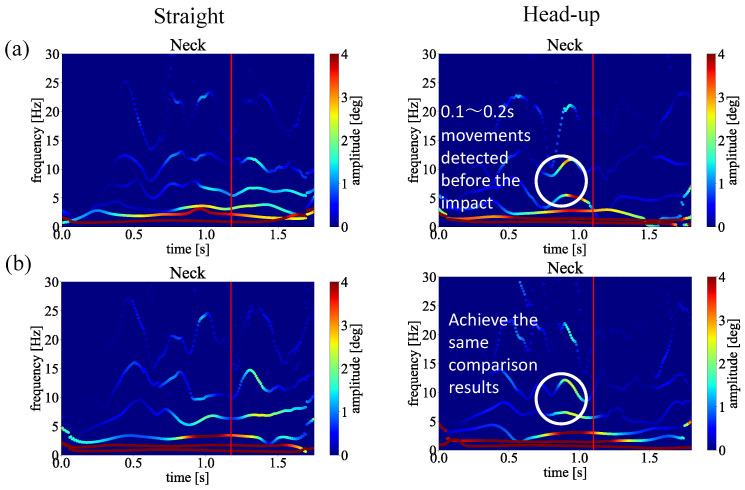
The HHT spectrum analysis of the average golfer by comparing straight and head-up golf swing motions. (**a**) Hip, Neck, Left Arm, Right Arm, Left up Leg, Right up Leg, Left Leg, and Right Leg joints as the input. (**b**) Hip and Neck as the input.

Next, to verify our proposed framework with sensitivity analysis, we changed our input joints, as shown in Figure 16. Since the MEMD has the filter bank property, the input joints may change the shape of the spectrum.

Figure 16a indicates the straight and head-up comparison we discussed in Figure 8 and Figure 9. Figure 16b shows the spectra obtained from our proposed framework that changed the input joints. As we can see from the two comparisons, the same results could be obtained even if the input joints changed the shape of the spectrum a little. Thus, our sensitivity analysis shows that our proposed framework could function correctly for different golf swing motion capture, focusing on biomechanical analysis.

## 5. Discussion

In this section, we discuss the above experimental results. First, compare the difference between straight trajectory swings and slice trajectory (head-up) swings from Figure 8 and Figure 9, focusing on these two spectra of the neck, for straight trajectory swings, high amplitudes are distributed around the timing of the impact. In golf swings, it is ideal that the greatest force is applied at the timing of the impact. In other words, it is good that there is no unnecessary movement before and after the impact and that the amplitude of the frequency increases (turns red) at the moment of impact. Therefore, in the straight trajectory swings, the high amplitude is distributed around the impact in all parts, so it can be considered that the amplitude was observed at the appropriate timing. On the other hand, in the slice trajectory (head-up) swings, it can be read from the spectra that high amplitudes are concentrated at the head (neck) before the timing of the impact.

This suggests that, in the case of Figure 10, the slice trajectory was caused by the head-up motion. In addition, when we compare the left lower body (LeftupLeg, LeftLeg), both have a large amplitude distribution around the impact. However, in the case of the slice, the amplitude is higher than that of the straight trajectory. In the case of the slice, the higher amplitude is less than that of the straight, indicating that the force is distributed. This may be due to the fact that the lower body is also affected by the heads-up motion.

Next, we consider the difference between straight trajectory swings and slice trajectory swings (body opening motion) from Figure 8 and Figure 10. Comparing the left lower body (LeftupLeg, LeftLeg), high amplitudes are concentrated prior to impact. In addition, since there is not much difference in the left arm (LeftArm), it can be seen that the cause lies in the left lower body (LeftupLeg, LeftLeg). In other words, in the case of Figure 10, it was possible to read from the spectra that the body opened before the impact and caused the slice trajectory. Also, when comparing the head (Neck), it can be seen that a high amplitude occurs around 0.4 [sec] in the slice trajectory swings. Since these amplitudes were not confirmed in the straight and slice (head-up) movements, it is thought that the movement of the neck was affected by the opening movement of the body.

In addition, for the single handicapper, our results demonstrate the same performance to detect unnecessary biomechanical motion causing the slice trajectory. To detect unnecessary biomechanical motion of the single handicapper, we indicate the spectra of straight trajectory in Figure 12a, and the spectra of slice trajectory in Figure 12b, respectively. As we can see from Figure 12a, almost no apparent modes with high amplitudes before the impact point have been observed. On the contrary, distinguished modes with higher amplitudes have been clearly observed in Figure 12b. As shown in Figure 12b, the single handicapper moved hip, right arm, and right leg joints before the impact time, by comparing to Figure 12a shows the golf swing motion that is a straight trajectory. As a result, the golf club had an angle toward the ball, as we mentioned in Section 2. By confirming these spectra, we point out which joint cause unnecessary biomechanical motion before the impact, causing the slice trajectory.

On the other hand, the beginner golfers present different biomechanical motions triggering the slice trajectory. As shown in Figure 13, Figure 14 and Figure 15, different golfers indicate different biomechanical motions before the impact points by comparing the spectra between straight and slice trajectories. These results revealed that, different from the average golfers and single handicappers with golf experience, these beginner golfers without any golf experience caused the slice trajectory by different joints in each swing. As a result, since we averaged the spectra for each straight and slice trajectory by several swings, the unnecessary biomechanical motions could not appear as clear as those obtained from the average golfer and single handicapper spectra. Thus, for beginner golfers, we need to adopt our method for each swing to support them in fixing the swing’s pose to achieve a straight trajectory.

In addition, the difference in biomechanical motions between golfers with experience years of golfing experience and the three beginner golfers also emerged after implementing our proposed method. On the one hand, Figure 8 shows the average golf spectra of the straight trajectory, and Figure 12a shows the single handicapper spectra of the straight trajectory. Both of them are experienced golfers with fixed forms. As shown in these figures, the white line indicates that the joint’s energy, the amplitude shown in the spectra, is symmetrically distributed around the time of impact. This reveals that experienced golfers perform golf swing motion efficiently from the point of view of biomechanics. On the other hand, Figure 13, Figure 14 and Figure 15 show the beginner golfer’s spectra of straight trajectory. Unlike the experienced golfers, the joint’s energy was asymmetrically and randomly distributed around the time of impact. This reveals that beginner golfers perform golf swings inefficiently due to their unfixed form.

The above results indicate that the HHT analysis of golf swing motions can accurately decompose unnecessary biomechanical motions, such as the head-up and open body at impact, and identify the causes of the slice trajectory. By identifying the causes, it is possible to apply the analysis to training support for golfers.

Furthermore, the results obtained by our research method can be employed in deep learning methods. For example, Kurbatskii et al. [42] proposed a method to forecast the non-stationary time series using HHT and neural networks. The decomposed modes can be considered training data and fed into neural networks for deep learning. Then, the feature works of this present research could be using spectra generated by our framework as features in the frequency domain to train neural networks that can automatically detect the head-up motion and body opening motion. In addition, other types of golf motions could be proposed based on our research. Other time series analysis methods also could be combined into our framework to improve the performance.

## 6. Conclusions

In this study, we focused on the head-up and open body at impact, often cited as the cause of slices by amateur golfers. We compared the difference between these movements and straight swing movements in the instantaneous frequency domain and examined them. In order to identify in the frequency domain the motions responsible for the straight and slice trajectories of the golf swing, inertial motion capture was adopted to quantify them. The collected data were evaluated from spectrum analysis and the biomechanical perspective using the Hilbert–Huang Transform. Thus, our conclusions of this study can be summarised as follows:

- The proposed golf swing analysis using HHT was able to identify the biomechanics that induces a golfer’s slice trajectory and straight trajectory in the instantaneous frequency domain.
- Our research revealed that spectrum analysis of head-up and body-opening movements could be applied to training support for golfers.
- After applying our method by analyzing golf swings captured from an average golfer, a single handicapper, and three beginner golfers, our method has been verified that it could help golfers to identify their biomechanical motions.
- Our method also presented IMF${}_{2-4}$ as parameters, that is, 0.1–0.2 s movements from 5 to 10 Hz frequency band, to help golfers identify their biomechanical motions.

In this study, golf swings were quantified using inertial motion capture and analyzed in the frequency domain using HHT to identify the cause of the slice trajectory from a biomechanical perspective. Although our proposed method could provide an easier and clearer way to identify biomechanical motions causing the slice trajectory by analyzing spectra, specialized knowledge about golf is still required. The future work could be considered as combining our method with other state-of-the-art methods, such as deep learning, to detect and extract biomechanical motions automatically since the extracted head-up and open body at impact with instantaneous frequency and amplitude could also be as trading data. Furthermore, using generative AI to develop a recommendation system that generates an individual-based optimized best swing to visually and intuitively teach the golfers to control the trajectory is another challenging task for future work.

## Figures and Tables

**Table 1:** The average golfer motion capture data details after synchronizing each type of trajectory to the same impact point for each group with 6 swings.

Swing	Time [s]	Frame Number	Impact Position
straight	1.93	231	144
slice: head-up	1.90	228	132
slice: opening	1.89	227	130

**Table 2:** The mean value and standard deviation with the coefficient of variation for instantaneous frequency were obtained by averaging six swings. The neck joint averaged data obtained from the straight swings were selected.

	Average	Golfer	Single	Golfer	Beginner	1	Beginner	2	Beginner	3
IMF	Mean ± SD	CV	Mean ± SD	CV	Mean ± SD	CV	Mean ± SD	CV	Mean ± SD	CV
1	31.57 ± 12.45	0.39	26.88 ± 10.93	0.41	36.53 ± 13.31	0.36	27.70 ± 11.10	0.40	34.52 ± 9.28	0.27
2	22.48 ± 3.64	0.16	21.02 ± 4.71	0.22	24.56 ± 3.61	0.15	20.15 ± 3.16	0.16	22.59 ± 3.23	0.14
3	11.58 ± 3.29	0.28	10.23 ± 4.72	0.46	14.23 ± 2.30	0.16	12.16 ± 1.63	0.13	12.73 ± 2.28	0.18
4	6.73 ± 2.37	0.35	5.20 ± 2.50	0.48	7.48 ± 1.45	0.19	6.15 ± 1.58	0.26	6.63 ± 1.15	0.17
5	3.65 ± 0.92	0.25	3.50 ± 1.58	0.45	4.16 ± 1.41	0.34	3.04 ± 1.04	0.34	4.06 ± 1.39	0.34
6	2.35 ± 0.57	0.24	2.16 ± 0.52	0.24	2.06 ± 0.58	0.28	1.96 ± 0.69	0.35	1.45 ± 0.59	0.41
7	1.30 ± 0.55	0.42	0.94 ± 0.82	0.87	1.22 ± 0.95	0.78	0.62 ± 0.30	0.48	1.28 ± 1.14	0.89

## Data Availability

The datasets generated and analyzed during the current study are available from the corresponding author upon reasonable request.

## Abbreviations

The following abbreviations are used in this manuscript:

HHT	Hilbert–Huang transform
HT	Hilbert transform
FT	Fourier transform
EMD	Empirical mode decomposition
MEMD	Multivariate empirical mode decomposition
IMF	Intrinsic mode function
BVH	Biovision Hierarchy
